# Transition Planning For After Polio Eradication

**DOI:** 10.1093/infdis/jix026

**Published:** 2017-07-01

**Authors:** Paul D. Rutter, Alan R. Hinman, Lea Hegg, Dennis King, Stephen Sosler, Virginia Swezy, Ann-Lee Hussey, Stephen L. Cochi

**Affiliations:** 1 Polio Eradication, World Health Organization, and; 2 Gavi, the Vaccine Alliance, Geneva, Switzerland;; 3 Task Force for Global Health, Decatur, Georgia;; 4 Global Immunization Division, Centers for Disease Control and Prevention, Atlanta, Georgia;; 5 Polio Team, Bill and Melinda Gates Foundation, Seattle, Washington;; 6 Immunization Team, United Nations Children’s Fund, New York, New York; and; 7 Rotary International, Evanston, Illinois

**Keywords:** Polio eradication, transition planning, immunization policy.

## Abstract

The Global Polio Eradication Initiative (GPEI) has been in operation since 1988, now spends $1 billion annually, and operates through thousands of staff and millions of volunteers in dozens of countries. It has brought polio to the brink of eradication. After eradication is achieved, what should happen to the substantial assets, capabilities, and lessons of the GPEI? To answer this question, an extensive process of transition planning is underway. There is an absolute need to maintain and mainstream some of the functions, to keep the world polio-free. There is also considerable risk—and, if seized, substantial opportunity—for other health programs and priorities. And critical lessons have been learned that can be used to address other health priorities. Planning has started in the 16 countries where GPEI’s footprint is the greatest and in the program’s 5 core agencies. Even though poliovirus transmission has not yet been stopped globally, this planning process is gaining momentum, and some plans are taking early shape. This is a complex area of work—with difficult technical, financial, and political elements. There is no significant precedent. There is forward motion and a willingness on many sides to understand and address the risks and to explore the opportunities. Very substantial investments have been made, over 30 years, to eradicate a human pathogen from the world for the second time ever. Transition planning represents a serious intent to responsibly bring the world’s largest global health effort to a close and to protect and build upon the investment in this effort, where appropriate, to benefit other national and global priorities. Further detailed technical work is now needed, supported by broad and engaged debate, for this undertaking to achieve its full potential.

The Global Polio Eradication Initiative (GPEI) was founded in 1988 [1] and has become a major global program over the succeeding 28 years. In 1988, an estimated 350000 children were paralyzed by polio and there were 125 polio-endemic countries [2]. In 2016, <50 children were paralyzed by wild poliovirus, and there were just 3 polio-endemic countries [3]. Despite complex challenges, setbacks, and delays [4] the program is close to achieving its goal.

This program built to eradicate polio now operates in >60 countries [5]. It delivers 2.2 billion doses of oral polio vaccine to 430 million children every year. It detects and investigates 100000 cases of acute flaccid paralysis annually. It tests stool samples from these cases—and from environmental surveillance sites—in a network of 146 laboratories, in 92 countries. It deploys >50000 community mobilizers and uses millions of volunteers in the hardest-to-reach and most-insecure communities to promote vaccine acceptance and health-seeking behaviors. The program employs >30000 personnel and millions of volunteers.

As fewer countries have active polio transmission, the GPEI is already in the process of ramping down its funding (Figure 1) and will close soon after polio eradication has been certified. As the program nears its end, a crucial question arises: what should happen to the assets, capabilities, and lessons of the GPEI?

**Figure 1.**
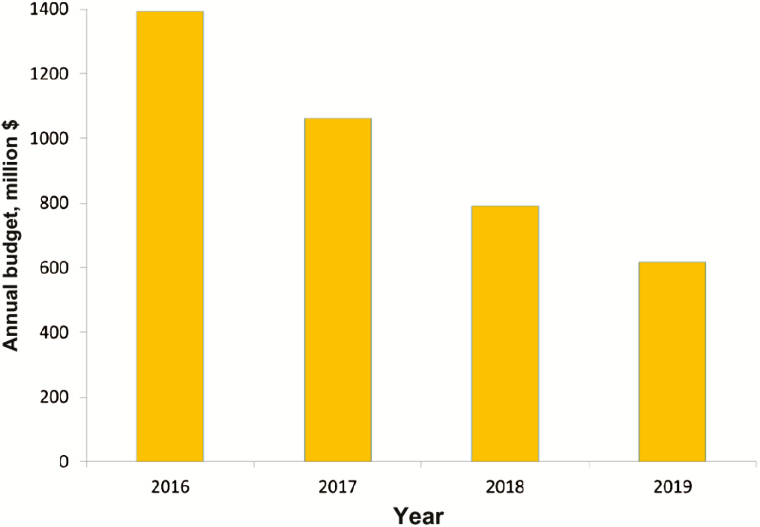
The budget of the Global Polio Eradication Initiative will reduce substantially as certification of polio eradication approaches [38].

The tools and functions that are currently funded by the GPEI, in whole or in large part, include millions of community-based health workers, social mobilisers, and volunteers; thousands of skilled staff at local, regional, and global levels; a global surveillance system and network of 146 polio laboratories; and a vaccine supply and logistics network. This global infrastructure has energized and mobilized communities; delivered high-quality interventions en masse; mobilized financial, political, and social support; responded to disease outbreaks and supported humanitarian emergencies; and created and maintained surveillance systems. In addition to their direct polio functions, GPEI staff also are involved in other activities, particularly relating to immunization services and surveillance of vaccine-preventable diseases.

There are significant risks. First, there is a risk that the polio-free world will not be sustained. Mitigating this requires that essential polio-related functions (eg, surveillance and outbreak response capacity) are sustained beyond certification. Second, there is a risk that other programs and health priorities, which, to varying degrees, have become reliant on GPEI-funded infrastructure for support, may suffer. More positively, the end of polio also creates significant potential opportunities for the capabilities and infrastructure built up by the GPEI to be redeployed or integrated to support other health priorities.

The GPEI is leading a detailed transition planning effort. This aims to mitigate the risks and to explore the potential opportunities associated with completing the eradication of polio. It also intends to ensure that the conclusion of the GPEI occurs carefully and responsibly. This article describes that effort in overview. Associated articles in this supplement describe important elements of it in greater detail. They build on work that has previously been described [6, 7].

## TRANSITION PLANNING GOALS

The GPEI is currently guided by the 2013–2018 Polio Eradication and Endgame Strategic Plan [8]. The last of the plan’s 4 objectives is to “ensure that the investments made to eradicate poliomyelitis contribute to future health goals, through a program of work to systematically document and transition the knowledge, lessons learned and assets of the [GPEI]” [8]. This program of work was initially called legacy planning, and is now referred to as transition planning. Transition planning needs to achieve 3 goals: (1) maintain and mainstream polio-essential functions after eradication has been certified, to protect a polio-free world; (2) where feasible, desirable, and appropriate, transition the capacities, processes, and assets that the GPEI has created to support other health priorities; and (3) capture and disseminate the lessons of polio eradication.

### Goal 1: Maintain and Mainstream Polio-Essential Functions After Eradication Has Been Certified, to Protect a Polio-Free World

Global polio eradication can only be certified when, with quality surveillance in place, at least 3 years pass without a detection of wild poliovirus. So 2020 is the earliest that certification could occur. Certification will be a major global milestone and achievement, but it does not mark a point at which all polio-related activity can cease. Three major polio-related functions will continue to be required: (1) immunization, (2) containment, and (3) outbreak detection and response. With regard to the first function, type 1 and type 3 oral polio vaccine will need to be withdrawn from use worldwide—just as type 2 oral polio vaccine was withdrawn in April 2016, following certification of type 2 wild poliovirus eradication in September 2015 [9]. For containment, even after certification, both wild and vaccine poliovirus strains will continue to be held in laboratories and in vaccine-producing facilities. A program of work is already underway to reduce the number of such facilities and to ensure that the facilities still holding virus do so safely. In terms of function 3, after polio eradication has been certified, the world needs to retain the ability to detect and respond to any case of polio that might occur. GPEI has built global networks of acute flaccid paralysis and environmental surveillance. After certification, these functions will continue to be required—perhaps in altered form. In September 2016, the GPEI started the process of developing a post–polio-certification strategy. This will specify in greater detail the polio-essential functions that will be needed after certification. To the degree possible, the intention is that the delivery of these functions should be integrated within mainstream immunization, containment, surveillance, and outbreak response systems.

### Goal 2: Where Feasible, Desirable, and Appropriate, Transition the Capacities, Processes, and Assets That the GPEI Has Created to Support Other Health Priorities

The facilities and transport functions of the Global Polio Laboratory Network do not only handle polio samples. Polio vaccinators do not only distribute polio vaccine. Among other things, they also distribute vitamin A, which is estimated to have prevented 1.5 million deaths since 1988 [10]. In one survey, staff funded by the GPEI estimated that they spend approximately half of their time working exclusively on polio eradication (Table 1). They spend the other half of their time working on routine immunization, measles and rubella control, new vaccine introduction, and a variety of other health areas. These data need additional study and verification but suggest a substantial risk to these other program areas as the funding of these staff progressively decreases.

**Table 1.** Estimated Time Allocation of Staff Funded by the Global Polio Eradication Initiative, 2014–2015 Survey

Activity	Percentage of Time
Polio eradication	46
Routine immunization	22
Measles and rubella prevention	8
New vaccine introduction	4
Child health days or weeks	4
Maternal, newborn, and child health and nutrition	5
Health systems strengthening	4
Sanitation and hygiene	2
Natural disasters and humanitarian crises	1
Other diseases or program areas	4

Contents of this table originally appeared elsewhere [37].

Other programs are variably aware of the risk that the closure of GPEI represents. GPEI has contributed to efforts to eliminate measles and rubella [11]. The Measles and Rubella Initiative estimates that 80% of its surveillance costs are funded in-kind by the GPEI [12]. Likewise, GPEI-funded staff have supported the planning, implementation, and monitoring of numerous supplementary immunization activities, as well as strengthening routine immunization systems [13], and a 2016 midterm review of the Global Vaccine Action Plan emphasizes that “all countries should mitigate any risk to sustaining effective immunization programs when polio funding decreases” [14].

Alongside the need to manage these risks, there is the potential for other health programs to take over some of the capacities, strategies, processes, and assets of the GPEI to positive effect. To date, the assets of greatest interest have included surveillance networks; laboratory networks and operational capacity; microplanning, which maps tens of thousands of entire communities in detail; social mobilization networks; data systems; and accountability mechanisms built between government and partners at national, state, district, and subdistrict levels. Capabilities have also been built at global and regional levels—such as the expert groups that have certified polio eradication from the regions, which could be repurposed to verify the elimination of measles virus and potentially other pathogens [15].

To date, discussion has focused particularly on the opportunities and risks for immunization and vaccine delivery programs and goals. There is interest in the relevance for global health security, and the potential to build on the polio surveillance and outbreak response systems as countries strengthen their capacity to meet their obligations under the International Health Regulations. There is also interest in understanding the wider potential for GPEI-funded assets to contribute to health systems strengthening and to the achievement of the Sustainable Development Goals.

Not all GPEI-funded assets and systems will be appropriate to transition. The GPEI transition planning process aims to facilitate the due diligence required to determine where such transitions are worth investing in. It also aims to ensure that, where GPEI investments will be discontinued, the process is done gradually and responsibly so that local health systems are not negatively affected.

### Goal 3: Capture and Disseminate the Lessons of Polio Eradication

This third goal is to ensure that the knowledge generated during nearly 30 years of polio eradication is harnessed, where relevant, in support of other public health goals—and that the lessons learned are shared. The positive lessons from the GPEI are many and varied (Table 2).

**Table 2.** Positive Lessons Learned: 10 Major Elements [7]

Communications and community engagement: mobilizing social and community support for vaccination
Communications and community engagement: using targeted disease initiatives as a springboard for broader health communication
The value of an advanced, state-of-the-art global, regional, and national laboratory network
Real-time disease surveillance and outbreak response capacity, data analysis, and immunization program monitoring
Addressing strategy implementation in conflict-affected areas and the risks of international spread to previously polio-free countries
Essential need for a program of research and innovation
Partnership coordination, advocacy, and resource mobilization
Strategic planning and policy development
Oversight and independent monitoring and evaluation
Monitoring of program accountability and performance

Some of these lessons are discussed in greater depth elsewhere in this supplement. The eradication goal—by its very nature—has required the GPEI to operate everywhere and anywhere. It would not be feasible to stop or suspend the program in difficult circumstances, such as those created by conflict or other humanitarian emergencies. The GPEI has had to learn how to operate effectively in these environments and can share these lessons with other programs [16, 17]. Similarly, the GPEI has used behavior and communications science with increasing sophistication and has lessons to offer from its work to make human behavior central to the eradication effort [18].

The lessons are not all positive—there are areas in which other programs could improve upon the GPEI’s approach. These include: closer collaboration with other programs, particularly immunization programs; setting appropriate targets; and using uniform definitions to always enable comparisons between countries. For other programs to benefit fully, both the positive and the negative lessons need to be documented and shared.

The lessons learned from polio eradication should be taken into account during the transition planning process detailed in the next section of this article. The GPEI has also launched the History Project, to record and retain the GPEI’s history. Work is underway to record specific technical best practices in a format that can be of direct use to other programs, and further such work is planned.

## TRANSITION PLANNING PROCESS

The 3 goals described above are those stated in GPEI’s 2013–2018 Eradication and Endgame Strategic Plan. GPEI’s primary responsibility is to organize and facilitate the process through which these goals can be achieved. GPEI alone cannot achieve these goals. This will require the active participation of governments, of programs other than polio and senior management within the 5 core agencies of GPEI, and of programs and stakeholders at national, regional, and global levels.

### Country Planning

Countries are asked to take the lead in transition planning, with the GPEI and other partners supporting. For this to succeed, the 3 goals of transition planning need to be considered in tandem with each country’s broader health needs and objectives. GPEI has published transition guidelines to advise ministries of health on this process [19]. These were developed following an examination of other large-scale change management and transition efforts in both private business and the global health sector (specifically, the US President’s Plan for Emergency AIDS Relief transitions, World Food Program sustainability programs, and transition of the Avahan HIV prevention program to the Government of India). The guidelines suggest that countries follow a 6-step process (Table 3). Experience drawn from the previous transitions indicates the importance of successfully completing each step sequentially.

**Table 3.** Country-Level Transition Planning: Summary of the 6-Step Process

Step	Key Milestone
Awareness raising	Appropriate members of government leadership are aware of the transition planning process and have an understanding of the GPEI footprint in their country, as well as ramp-down projections
Coordination	A governing body and coordination/management team is identified, with a high-level work plan established
Evidence	A complete map of polio assets, accurate budget ramp-down data, country health priorities, and needs mapping is presented
Strategic options	Conduct a transition planning workshop/ simulation exercise with a broad group of stakeholders
Vision for the future	Draft transition plan/business case is shared with stakeholders for input
Roadmap	Jointly agreed strategy for moving forward is developed, with funding commitments and execution roadmap

Contents of this table originally appeared elsewhere [19].

**Figure 2.**
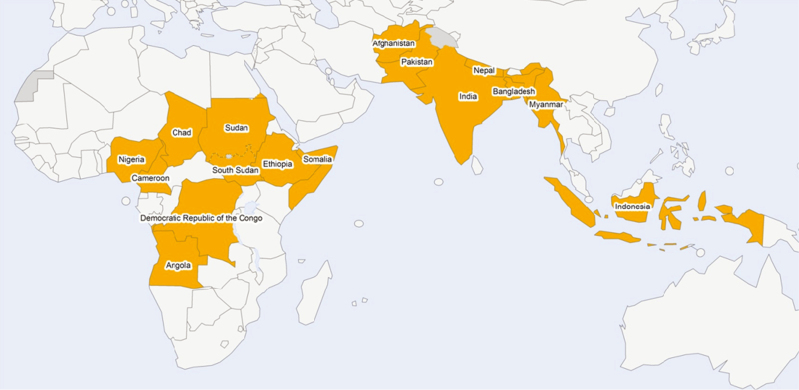
The 16 priority countries for polio transition planning [8].

More than 60 countries receive funding from GPEI. All are encouraged to undertake transition planning. The need is particularly pressing for 16 of these countries, which collectively benefit from >95% of GPEI’s total personnel funding worldwide. GPEI has designated these as priority countries [8] (Figure 2) and is focusing particular attention on supporting their transition planning processes.

Of these countries, India is the furthest advanced in developing a transition plan. That plan puts particular emphasis on repurposing the surveillance and social mobilization networks built by the polio program [20].

A number of countries that stopped polio transmission some years ago are already well advanced in the process of adapting their polio programs. Their ideas and experiences may be of interest to others. In Nepal, for example, the polio surveillance system expanded to include measles and neonatal tetanus in 2003 and to Japanese encephalitis and rubella in 2004 [21]. GPEI-funded staff have also been centrally involved in new vaccine introduction and in responding to other outbreaks. An important issue for these countries is to sustain funding for key functions that contribute to strengthening immunization and other health services as GPEI winds down. Other countries that are no longer supported directly by GPEI also offer lessons, such as China’s expansion of its polio and measles surveillance networks, using a similar approach to that of Nepal [22].

In Nigeria, the value of developing a strong transition plan was highlighted by the country’s 2014 experience with Ebola. When Ebola virus was detected in Nigeria, the emergency operations center capability that had been put in place to manage the polio program was quickly repurposed to manage the Ebola outbreak, which was quickly terminated. GPEI-funded assets are also working to strengthen routine immunization in Nigeria [23]. The National Stop Transmission of Polio program, for example, was created in 2012 with the support of the Centers for Disease Control and Prevention and now involves >180 officers posted across the country. In addition to polio surveillance and outbreak response, their work also helps to strengthen measles surveillance, routine vaccination coverage, and outbreak response for other diseases [24].

The other priority countries have also started to map their GPEI-funded assets and systems and to consider how they might be integrated into national health systems. In Cameroon, there is particular interest in how the detailed maps and microplanning developed to stop polio transmission can be used for other purposes [25]. In South Sudan, the earliest interest is in how to repurpose the National Stop Transmission of Polio program [26].

### Agency Planning

The GPEI has 5 core partner agencies: the Bill and Melinda Gates Foundation, the Centers for Disease Control and Prevention, Rotary International, the United Nations Children’s Fund, and the World Health Organization. Each is also developing a robust process to plan for post-polio eradication transition. The 5 agencies are coordinating as appropriate as they do so. For the World Health Organization and the United Nations Children’s Fund, which manage the majority of personnel supported by GPEI funding, agency-specific plans are critical for managing organizational change as the GPEI winds down and for providing appropriate guidance to GPEI-supported personnel. For all GPEI partners, this process will ensure that the agency considers the risks and opportunities that the end of GPEI presents for its other program areas and the lessons offered by its experience. As an important example, Rotary International was instrumental in establishing the global eradication goal, and its PolioPlus program offers a particular set of lessons, described elsewhere in this supplement [27].

This agency planning also needs to ensure that the global and regional level capabilities developed by GPEI are sustained and developed as appropriate. The Global Polio Laboratory Network is an important example. It has already been expanded to include other vaccine-preventable diseases and has the potential to be expanded further [28, 29].

There will naturally be some overlap between the program areas that will be considered at the country level and those that will be considered by the agencies. The major areas are as follows: how can and should the surveillance networks that are centered on acute flaccid paralysis be expanded systematically for other purposes, particularly for surveillance of vaccine-preventable diseases? [30] What has been the impact of the Stop Transmission of Polio program, and what should its future entail? [31] And how can the social mobilization networks that have been built to engage communities in eradicating polio be most effectively repurposed? [32] Where necessary, the GPEI aims to facilitate this dialogue—so that the agencies’ position is known to countries and so that countries’ views and plans are taken into account in the agencies’ global and regional level planning.

### Oversight, Management, and Monitoring

The director-general of the World Health Organization, the executive director of the United Nations Children’s Fund, the president of Global Development of the Bill and Melinda Gates Foundation, the president of Rotary International, and the director of the Centers for Disease Control and Prevention collectively form the Polio Oversight Board. This board oversees the implementation of the GPEI, including transition planning.

The work of the GPEI is led by a series of management groups. These include members from each of the 5 core GPEI agencies and others, as relevant. In 2013, the Strategy Committee established a Legacy Management Group (now the Transition Management Group) [33] to oversee the implementation of this work.

The GPEI has an Independent Monitoring Board, which monitors and guides progress toward poliovirus interruption globally [34]. Based on that model but operating separately, the Polio Oversight Board has established the Transition Independent Monitoring Board. This board will monitor and guide the development and implementation of transition plans in countries and help to ensure that all necessary stakeholders are being involved in the process. Following a preparatory meeting in November 2016, the board will meet every 6 months and issue independent reports on progress.

## CONCLUSIONS

The need for transition planning (then called “legacy planning”) was first highlighted by the GPEI’s Independent Monitoring Board in 2012 [35], and the 2013 Polio Eradication and Endgame Strategic Plan signaled a serious intent to address this. But poliovirus transmission had not yet been stopped worldwide, and many viewed transition planning as premature. In late 2015 and into 2016, this view has shifted. In January 2016, the Polio Oversight Board agreed that each of the core GPEI agencies should develop its own transition plan [36]. In May 2016, the GPEI published a 4-year budget that made real the significant year-on-year budget decreases that will occur between 2017 and 2019. Between March and July 2016, the staff time allocated to transition planning by the GPEI partners increased 3-fold. By November 2016, GPEI had established the Transition Independent Monitoring Board to help accelerate focus on this work. By the end of 2016, there were some positive signs of countries engaging in transition planning—but this progress was variable.

There is not widespread understanding or agreement on the true extent of the risks and opportunities that the end of the polio program entails. It would be helpful for those whose programs will be affected to commission independent assessments of this—for example, immunization stakeholders, including Gavi, might usefully assess the impact on immunization systems in key countries.

Polio transition planning is a complex area of work—with difficult technical, financial, and political elements. There is no significant precedent. There is forward motion and willingness on many sides to understand and address the risks and to explore the opportunities. Very substantial investments have been made over 30 years to eradicate a second human pathogen from the world. Transition planning represents a serious intent to protect and build on these investments. Further detailed technical work is now needed, supported by broad and engaged discussion, for this undertaking to achieve its full potential.

## Notes

***Disclaimer.*** The findings, interpretations, and conclusions expressed in this article are those of the authors and do not necessarily reflect the policies or views of the United Nations Children’s Fund, the World Health Organization, Gavi, the Task Force for Global Health, the Bill and Melinda Gates Foundation, Rotary International, or the Centers for Disease Control and Prevention.

***Supplement sponsorship.*** This work is part of a supplement coordinated by the Task Force for Global Health with funding provided by The Bill and Melinda Gates Foundation and the Centers for Disease Control and Prevention.

***Potential conflicts of interest.*** All authors: No reported conflicts. The authors have submitted the ICMJE Form for Disclosure of Potential Conflicts of Interest. Conflicts that the editors consider relevant to the content of the manuscript have been disclosed.
